# Health Literacy as a Potential Determinant of Healthcare Use Among Young Adults in German‐Speaking Countries With a Special Emphasis on Primary Care: A Scoping Review

**DOI:** 10.1111/hex.70945

**Published:** 2026-11-04

**Authors:** Anna‐Sophia Beese, Valentin Leu, Oliver Senn, Saskia Maria De Gani

**Affiliations:** ^1^ Careum Foundation, Center for Health Literacy Zurich Switzerland; ^2^ Institute of Primary Care, University Hospital Zurich University of Zurich Zurich Switzerland; ^3^ Department of Health Careum School of Health, Kalaidos University of Applied Science Zurich Switzerland

**Keywords:** digital health, primary care, health literacy, healthcare utilisation, mental health, primary care, young adults

## Abstract

**Background:**

In recent years, the increasing number of medical consultations among young adults in German‐speaking countries, particularly in general practice, has attracted growing attention from media and healthcare providers. The possible reasons remain speculative. However, the consequences for the system—higher workforce load and costs, among other things—are probable. Nevertheless, little scientific research has investigated the underlying causes of this trend. Initial hypotheses from the Swiss Medical Association suggest that limited health literacy, increasing uncertainty in managing personal health amid digital information overload, and a decline in mental health may be contributing factors. Thus, this study aimed to explore these hypotheses scientifically, focusing on the potential link between health literacy and healthcare utilisation among individuals aged 18–35 years.

**Methods:**

This study undertakes a scoping review following the PRISMA‐ScR (PRISMA extension for scoping reviews) framework. The review examined studies addressing the increase in medical consultations among young adults in Switzerland, Germany and Austria, and explored the proposed causes of this trend, and the role of health literacy in shaping health behaviours in this age group. The online databases PubMed, Embase, CINAHL, Cochrane Library, Web of Science were used to identify studies published between January 2005 and July 2025, focusing on individuals aged 18–35 and conducted in Switzerland, Germany or Austria, preferable with a research focus on primary care.

**Results:**

25 studies met the inclusion criteria. The analysis revealed a fragmented evidence base regarding the drivers of increased healthcare utilisation in this population. While topics such as mental health burdens, social media influence, healthcare system strain, and associations with health literacy are discussed, robust empirical data remains scarce.

**Conclusions:**

The findings highlight key research gaps and the need for further studies to understand multifactorial causes of increased healthcare use among young adults. They provide a foundation for developing evidence‐based strategies to improve health literacy and support needs‐based healthcare utilisation.

**Patient or Public Contribution:**

The review was conceptualised and conducted by an interdisciplinary research team and lived experience researchers in general internal medicine.

## 1 Introduction

Over the past decade, the number of people seeking medical care in Switzerland has steadily increased [1]. Stratified data from the Swiss health authority report indicate a notable decline in the proportion of younger adults who did not consult a medical doctor (including both general practitioners and specialists) within a given year. Among individuals aged 25–34 years, this proportion decreased from 28% in 2012 to 21% in 2022, while among those aged 15–24 years it declined from 24% to 20% over the same period [1].

Furthermore, datafrom the Swiss Medical Association (FMH) show that the average number of patients per medical practice exceeded 1000 for the first time in 2022, marking a 15% increase compared to 2017, with a particularly sharp 6.5% rise between 2021 and 2022 (Quelle 1). The Swiss health insurer Sanitas further reports that among adults aged 26–35, the proportion of individuals who did not use any basic insurance‐covered healthcare services dropped from approximately 35% in 2012 to around 22% in 2022 [2, 3]. The healthcare utilisation of young adults thus represents a significant driver of the increasing workload and economic consequences in general practices in Switzerland.

In newspaper interviews, healthcare professionals attribute this development to greater health concerns and lower tolerance for diagnostic uncertainty among young adults as well as the influence of social media and increased digital information seeking behaviour [2, 4]. Trends that might have been exacerbated by the COVID‐19 pandemic and the proliferation of online health information [3, 5, 6]. Although digital media have substantially increased access to health‐related information, greater information availability does not necessarily result in higher levels of health literacy. Individuals often face challenges in identifying trustworthy information, critically evaluating its quality, and applying it appropriately to their own health decisions [7]. General practitioners (GPs) further report that even minor symptoms frequently prompt patients to request extensive diagnostic testing, reflecting growing uncertainty and anxiety about health conditions [2, 4]. Moreover, physicians observe a loss of basic self‐care knowledge and unrealistic expectations regarding rapid recovery, which could lead to earlier and more frequent consultations by young adults [2].

This shift towards an increased healthcare utilisation has also intensified cost pressures [6, 8]. According to the FMH, rising first‐time consultations are a central contributor to escalating healthcare expenditures, with young adults accounting for a disproportionate share of this growth [6]. Concurrently, primary care providers face increasing workloads and stress, which limits consultation time per patient and raises concerns about the quality of care delivered [2, 9]. In line with the reports from Switzerland, health professionals in the neighbouring countries Germany and Austria report increases in healthcare utilisation by young adults in the primary care sector. German studies further report high case numbers and noticeable bottlenecks in GP's practices; regarding young adults, mental health services in particular show increasing use [10, 11]. In Austria, too, high usage of healthcare services in primary care among young adults is well documented and findings regarding waiting times and admission freezes indicate an overburdening of primary care [12, 13].

These findings are consistent with other European data showing that demand for primary care since the COVID‐19 pandemic—including primary and mental healthcare—has increased, indicating lasting changes in younger people seeking medical help [11]. A study from Norway, for example, emphasises the substantial increase in GP consultations for mental diagnoses and conversational therapy among youth and young adults between 2006 and 2021 [14]. Further possible reasons for an increase in healthcare utilisation commonly cited by international studies are an increase in mental morbidity and psychosocial issues among young adults (e.g., depression/anxiety, stress, life transitions) [14, 15] or a low‐threshold access through telephone/online channels facilitating initial and follow‐up contacts [16, 17].

Overall, these results highlight a complex interplay of behavioural, socioeconomic and systemic factors potentially contributing to an observable increased healthcare utilisation among young adults in Switzerland and various European countries, with significant implications for healthcare costs, provider workload, and the quality as well as sustainability of primary care delivery.

However, the proposed potential drivers of this trend, including difficulties in dealing with health‐related information and increased health‐related uncertainties, the influence of social media and digital information, and the impact of the COVID‐19 pandemic [3, 4, 11], have remained largely unexamined. One concept which could be important in this context is health literacy (HL), i.e., people's ability to find, understand, appraise, and apply health information to make appropriate health‐related decisions [18]. With regard to more shared decision‐making approaches and more participation of patients and their relatives in healthcare [19], as well as calls for more sensible and appropriate use of healthcare services, a high level of HL is crucial. HL enables people to understand health‐related information, to appropriately navigate the healthcare system, to weigh treatment options, to engage effectively in self‐management and to strengthen their own and others health and well‐being [19]. Importantly, HL is not solely an individual attribute but is shaped by organisational and broader systemic factors. An “HL‐responsive” healthcare system—one that reduces complexity, provides accessible, comprehensible information and care processes and empowers patients—is essential to support people in navigating and using healthcare services effectively [20, 21]. Beyond individual competencies, organisational health literacy (OHL) emphasises the responsibility of healthcare organisations to make health information, communication, and services easier to access, understand, navigate, and use. Health‐literate organisations can facilitate appropriate healthcare utilisation by reducing organisational barriers, particularly for populations with limited health literacy, including young adults. Thus, healthcare utilisation is shaped not only by individual health literacy but also by the responsiveness of healthcare organisations to patients' health literacy needs [22, 23]. Evidence from Switzerland and other European countries demonstrates that low HL is associated with poorer health outcomes and increased healthcare utilisation [7, 24]. Individuals with limited HL often struggle with interpreting health‐related information accurately, leading to suboptimal decision‐making, inefficient healthcare use, and higher system costs [24].

Given these developments of increased healthcare utilisation, there is a pressing need to better understand the interplay between HL, related determinants and the healthcare utilisation among young adults in German‐speaking countries with a special emphasis on Switzerland. Thus, as a first step, a scoping review was conducted in order to systematically map the research in this area, as well as to identify any existing gaps in knowledge.

The aim of this scoping review was to map and characterise the current state of evidence on the healthcare utilisation of young adults (aged 18–35) in German‐speaking countries, to identify gaps in research, and to explore potential explanatory factors for the increased healthcare utilisation, in particular in primary care, but also in other ambulatory settings.

The following research questions were formulated:

- **RQ1:** What studies are available to date that have investigated healthcare utilisation in primary care by young adults in Switzerland, Germany and Austria?
- **RQ2:** What causes for the increase in healthcare utilisation by young adults in Switzerland, Germany and Austria are discussed in the literature?
- **RQ3:** What correlations between HL and the utilisation of the healthcare system by the general population in Switzerland, Germany and Austria are reported in the literature?
- **RQ4:** Are there studies that have already investigated the role of HL on healthcare utilisation by young adults in Switzerland, Germany and Austria?

## 2 Methods

This scoping review was conducted using the framework developed by Arksey and O'Malley [25], a widely applied approach for such reviews [26]. The framework comprises five stages: (1) identifying the research question, (2) identifying relevant studies, (3) selecting studies, (4) charting the data, and (5) collating, summarising, and reporting the results. In addition, the present study followed the PRISMA‐ScR guidelines (**P**referred **R**eporting **I**tems for **S**ystematic Reviews and **M**eta‐**A**nalyses – Extension for Scoping Reviews) [27]. By providing a structured and standardised guideline, the application of PRISMA‐ScR ensures transparency and completeness in the reporting of scoping reviews.

The following inclusion and exclusion criteria were used to define the scope of this review (see also Table 1): Eligible studies were peer‐reviewed articles and reviews as well as reports, book chapters and grey literature with a focus on the increasing healthcare utilisation among young adults in the German‐speaking countries Switzerland, Germany and Austria (with a focus on primary care and potential determinants leading to this phenomenon. The original plan was for the study to focus primarily on Switzerland, as the empirical observations underlying the phenomenon under investigation were derived mainly from sources reflecting the experiences of Swiss healthcare professionals. However, in the very beginning of the research process, the research team recognised that, given the available data, it would be appropriate to broaden the scope to include the German‐speaking region. The cultural contexts as well as the healthcare systems of Switzerland, Germany, and Austria are highly comparable, allowing meaningful conclusions to be drawn regarding the situation across all three countries and beyond. Articles and reports were included if they were published between the period of January 2005 and July 2025, were written in English or German (due to the language capacities of the research team), focused on young adults aged 18–35 years and involved the healthcare utilisation in primary care and other ambulatory settings. A 20‐year time frame was selected to capture both landmark and longitudinal studies as well as recent evidence. Quantitative, qualitative, mixed‐methods designs and scientific reports were considered to include different research approaches on the topic. Articles were excluded if they did not fit into the conceptual framework of the study and these inclusion criteria.

**Table 1:** Inclusion and exclusion criteria.

Category	Inclusion criteria	Exclusion criteria
Type of publication	Peer‐reviewed articles, reviews, reports, book chapters, and grey literature	Publications not matching the specified publication types
Geographical focus	Studies conducted in or focusing on Switzerland, Germany, and Austria	Studies conducted outside Switzerland and German‐speaking countries
Topic	Studies addressing increasing healthcare utilisation among young adults and/or determinants influencing this phenomenon	Studies not related to healthcare utilisation or not fitting the conceptual framework of the review
Population	Young adults aged 18–35 years	Studies focusing on populations outside the defined age range
Healthcare setting	Healthcare utilisation in primary care and other ambulatory settings	Studies unrelated to healthcare utilisation in primary care or other ambulatory settings
Time frame	Publications published between January 2005 and July 2025	Publications outside the defined publication period
Language	Publications written in English or German	Publications written in other languages
Study design	Quantitative, qualitative, and mixed‐methods studies as well as scientific reports	Non‐academic study designs of low scientific quality
Conceptual relevance	Studies aligned with the conceptual framework of the scoping review	Studies not fitting the conceptual framework or inclusion criteria

Literature search was performed from October 2024 to July 2025 by two study team members. Publications of interest were identified by using controlled vocabulary terms (MeSH (medical subject headings) terms for PubMed/Medline and CINAHL Headings for CINAHL) and free‐text terms of keywords with Boolean operators (AND, OR), adapted for each database, including: “health literacy”, “competence for health”, “young adults”, “youth”, “doctor visits”, “visits to the doctor”, “healthcare surveys”, “healthcare utilisation”, “health behavio*”, “health service use*”, “Switzerland”, and “Swiss*”. The detailed search strategy can be found in the Supporting Information: Table S1. The following bibliographic databases were searched after a thematic scan of health/health sciences resources and consultation with an experienced librarian from the University Library: PubMed, Embase, CINAHL, Cochrane Library, Web of Science, and Medline. These databases were considered particularly relevant because they collectively provide comprehensive access to high‐quality, peer‐reviewed medical, biomedical, and interdisciplinary research, ensuring a broad and reliable foundation for evidence‐based studies. In addition, a hand search was conducted to identify relevant grey literature, including articles from newspapers, reports from official national health institutions (e.g., the Swiss Federal Office of Public Health (FOPH)), and additional material retrieved via Google Scholar. After duplicate removal, two reviewers (ASB and VL) independently screened all titles and abstracts against the predefined eligibility criteria. In a second screening step, the same two reviewers independently undertook full‐text screening of the remaining records in accordance with the predefined eligibility criteria by using Excel. In both screening steps, any discrepancies in record classification were resolved through discussion between the two reviewers. To support the manual full‐text screening, AI was used as a supplementary decision‐support tool. Following manual assessment by the reviewers, manuscripts for which there was uncertainty regarding their relevance to the four predefined research questions were additionally examined using ChatGPT and the AI‐based scientific literature tools Research Rabbit and Perplexity. The purpose of this step was not to automate study selection but to obtain an independent assessment of whether the manuscript addressed any of the review questions. The AI outputs were considered solely as supplementary information and did not determine study eligibility. All inclusion and exclusion decisions were made by the review team based on the predefined eligibility criteria, with any discrepancies resolved through discussion and consensus.

The review process identified 25 eligible studies. Data extraction was carried out independently by the two reviewers using a jointly developed Excel data‐charting form, which recorded bibliographic details (author, title, design/methods), sample characteristics (size, population/sample, country, and setting), substantive variables relevant to the research questions (utilisation outcomes), and key results (general, specific to review questions, key messages). The two reviewers independently charted the data of all identified studies, discussed the results and continuously updated the data‐charting form in an iterative process. Thus, data extraction results were discussed continuously, and any differences were resolved by consensus and discussion with other reviewers of the study team if needed. The final search results were exported into the reference management software Citavi, and duplicates were removed by a member of the study team.

The 25 documents included in the study were coded and analysed in depth with MAXQDA (VERBI Software 2024), a programme for qualitative data and text analyses. The articles and reports were systematically reviewed, and text passages were coded to identify matches with the research questions. Recurring themes and research priorities were identified through inductive coding during the full‐text analysis. Each study was assigned to one or more RQs based on its primary content and objectives. This thematic categorisation served to descriptively map the range and focus areas of current research and to identify thematic clusters as well as potential research gaps [25].

### 2.1 Analysis Framework for the Scoping Review

The data analysis for the scoping review was conducted on two complementary levels: descriptive mapping and analytical synthesis.

- **Level 1:** Descriptive mapping (core of the scoping review) focused on bibliographic information, study designs, research settings, outcomes, population/sample characteristics, and reported results. This information directly informed RQ1 (trend evidence: utilisation outcomes) and RQ3 (correlations between HL and utilisation associations).
- **Level 2:** Analytical synthesis (causal/conceptual framework): By analysing the identified studies in MAXQDA, the extracted findings were structured into explanatory themes—such as causes, moderators, and mechanisms—and organised along the four RQs. This approach enabled to explore the topic in depth and systematically address all four RQs (with a particular focus on RQ2 (determinants on different levels) and RQ4 (HL as mediator/moderator of *increase* among young adults).

## 3 Results

A total of 638 records were initially identified (see PRISMA‐ScR flow diagram, Figure 1, 507 were excluded at the first stage for not meeting the inclusion criteria, and 16 were removed as duplicates. During title and abstract screening from the remaining 115 titles, 63 records were excluded: 53 due to irrelevance of topic or sample, 3 because they were non–peer‐reviewed newspaper articles, and 7 for which no full text was available. The remaining 52 full‐text articles were then assessed for eligibility. Of these, 27 were excluded for the reasons documented (e.g., not relevant to the review question, insufficient methodological detail, or outside the defined timeframe). Ultimately, 25 records were included in the final synthesis.

**Figure 1:**
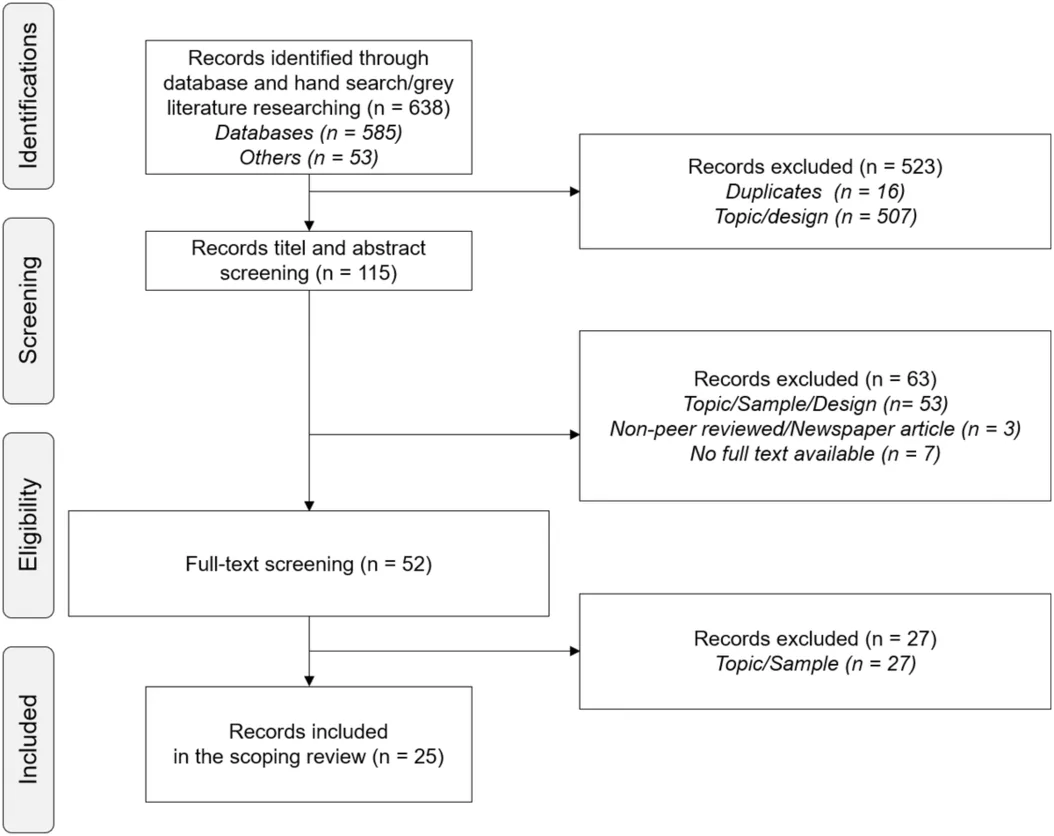
PRISMA‐ScR Flowchart.

### 3.1 Descriptive Mapping—Characteristics of Included Records

A detailed overview on the records included is presented in Table 2. Of the 25 included records, 21 were original research articles and four were reports. Most studies employed a cross‐sectional population‐based design. Eighteen publications (72%) were published in English and seven (28%) in German. Only four studies focused exclusively on young adults, while five (ID 5, 6 (mixed), 10, 12 (mixed) and 21) were conducted in general practice settings. Geographically, the evidence originated from Switzerland (*n* = 14), Germany (*n* = 8), and Austria (*n* = 3). Overall, the number of publications addressing health literacy and healthcare utilisation among young adults increased between 2019 and 2024.

**Table 2:** Overview of the included records.

ID	Authors	Title	Year	Country and setting	Study design/methods	Sample		Key results relevant to the scoping review	RQs
1c	Amstad, F.; Surís, J.‐C.; Barrense‐Dias, Y. et al.	*Original title in German:* Gesundheitsförderung für und mit Jugendlichen und jungen Erwachsenen: wissenschaftliche Erkenntnisse und Empfehlungen für die Praxis *Translated title in English:* Health promotion for and with adolescents and young adults: scientific findings and recommendations for practice	2022	Switzerland	Report	—		One fifth of young people experience a mental health crisis or mental illness by the age of 25 Potential causes for healthcare utilisation are an insufficient level of HL, an increase in (social) media consumption and, as a result, uncertainty among young adults caused by health influencers and information overload.	2,4
				Adolescents and young adults					
2	Bardy, T. L. C.	Assessing health insurance literacy (HIL) in Switzerland: first results from a measurement tool	2024	Switzerland (German, French, Italian regions)	Quantitative study, including ordinary least squares and quantile regressions	6036 adults		Lower HIL scores among young adults. Because younger individuals are healthier and in general fewer use healthcare services, they may perceive health insurance choices as less critical and, thus, report a lower HIL score.	3
				Assessing health insurance literacy in the general Swiss population					
3	Berens, E.‐M.; Vogt, D.; Messer, M.; et al.	Health literacy among different age groups in Germany: results of a cross‐sectional survey	2016	Germany	Quantitative cross‐sectional study	2000 participants aged 15 years or older		Of the respondents aged 15–29 years, 47.3% had limited perceived HL and 47.2% of those aged 30–45 years. For young adults, limited functional HL, low social status, a migrant background and a high number of doctor visits were associated with limited perceived HL. Among young adults, more than 70% of those with a high number of doctor visits are also at special risk of having limited perceived HL. Explanations: an increasing number and complexity of health problems at this age that is typically associated with many different life course decisions in the family, work and community area (‘rush hour of life’).	3,4
				Private households					
4	Berens, E.‐M.; Vogt, D.; Ganahl, K.; et al.	Health Literacy and Health Service Use in Germany	2018	Germany	Quantitative survey	2000 people in Germany (nationally representative sample)		Respondents with lower HL scores reported more frequent use of all four included types of curative health services. Multiple regression analysis showed a direct significant effect of HL on doctor (*β* = –0.066) and other health professionals visits (*β* = –0.103)	3
5	Bohlken, J.; Kostev, K.; Riedel‐Heller, S.; Hoffmann, W.; et al.	Effect of the COVID‐19 pandemic on stress, anxiety, and depressive disorders in German primary care: A cross‐sectional study	2021	Germany	Quantitative observational study based on cross‐sectional medical record data	3,021,042 patients who visited at least one out of 757 GP practices		The paper discusses several causes: Pandemic‐related stressors (social isolation, restrictions, home office, childcare responsibilities). Younger adults showed increasing diagnoses later in 2020, reflecting growing strain during the prolonged pandemic.	2
				GP practices					
6	Bundesamt für Statistik (BFS)	*Original title in German:* Schweizerische Gesundheitsbefragung: Anzahl Arztbesuche Schweiz: Anzahl Konsultationen bei Generalistinnen und Generalisten; Anzahl Konsultationen bei Ärztinnen und Ärzten *Translated title in English:* Swiss Health Survey: Number of doctors' visits in Switzerland: number of consultatnons of general practitioners; Number of consultations with doctors	2022	Switzerland	Quantitative	—		Increase in number of consultations of GPs in the last 12 months among young adults aged 15–24 years and 25–34 years in Switzerland.	1
7	De Gani, S.M.; Jaks, R.; Bieri, U.; et al.	Health Literacy Survey Switzerland 2019‐2021	2021	Switzerland	Quantitative national survey	2502		This survey provides representative Swiss data showing that limited HL is common and strongly associated with increased use of healthcare services (particularly GP visits, hospitalisations, and emergency care). It identifies socio‐demographic disparities as drivers and underscores HL as a structural factor influencing healthcare utilisation. While it does not analyse temporal increases in usage, it positions HL as a central explanatory variable.	3
				Adult residents (age ≥ 18 years) of Switzerland					
8	FMH	*Original title in German:* Die zunehmende Nachfrage nach ärztlichen Leistungen ist eine Herausforderung für die Ärzteschaft *Translated title in English:* The increasing demand for medical services is a challenge for the medical profession	2023	Switzerland	Report	—		The main reasons for the increase in healthcare utilisation are: a need for advice is increasing; a change in health awareness is emerging; uncertainty regarding the assessment of disease symptoms. The increase in the number of patients is not a phenomenon of 2022. However, the long‐term trend has been exacerbated by the COVID‐19 pandemic. Infections have increased since social distancing measures were stopped.	1
				General population					
9	Franzen, J.; Mantwill, S.; Rapold, R.; et al.	The relationship between functional health literacy and the use of the health system by diabetics in Switzerland	2013	Switzerland	Mixed methods	493 aged between 35–70 years old		Limited functional HL is associated with higher outpatient costs and more physician visits among adults with type 2 diabetes in Switzerland. While the study does not focus on young adults in particular or utilisation increases over time, it offers strong evidence for HL being a critical factor for healthcare utilisation and costs in the Swiss context.	3
				Insured persons of the basic health insurance plan of the largest health insurer in Switzerland					
10	Frese, T.; Klauss, S.; Hermann, K.; et al.	Children and Adolescents as Patients in General Practice ‐ The Reasons for Encounter	2011	Germany	Cross‐sectional analysis study	805 patients under 20 years old		Descriptive, cross‐sectional data on why children and adolescents (0–19) consult GPs in Saxony, Germany. Most visits are for common acute conditions or preventive care, with adolescents showing a wider spectrum including injuries and contraception.	3,4
				GP practices in Saxony, Germany					
11	Gerich, J.; Moosburger, R.; Heigl, C.	Health literacy and age‐related health‐care utilisation: a multi‐dimensional approach	2022	Austria (Upper Austria)	Quantitative survey	590 adults (18–80 years old)		This Austrian study does not address rising utilisation among young adults (RQ1) but provides strong evidence for RQ3 and RQ4, showing that HL profiles significantly shape utilisation patterns and amplify age‐related increases. It also links causes (RQ2) to HL‐related reliance on physicians and external locus of control. Some groups with “high HL” scores may not actually be empowered; instead, they may overly rely on physicians, accept prescriptions passively, and prefer paternalistic interactions. This leads to more frequent doctor visits and higher medication costs.	2, 3, 4
12	Giezendanner, S.; Bretschneider, W.; Fischer, R.; et al.	The ecology of medical care in Switzerland: prevalence of illness in the community and healthcare utilisation in Switzerland	2020	Switzerland	Population‐based, cross‐sectional health survey with a multistage, stratified cluster design	1025 (18–85 years old)		Swiss, pre‐pandemic baseline of where adults seek care. Age‐related utilisation patterns (notably an emergency department peak in 18–29 years old), while primary care carries the main load and should be resourced accordingly. Relevant system features: primary care capacity, specialist density, gatekeeping versus free provider choice, and supply‐sensitive consumption may shape overall utilisation levels.	2,3
				Adult Swiss population					
13	Grobe, T.G.; Weller, L.; Braun, A.; Szecsenyi, J.	BARMER Arztreport 2024	2024	Germany	Qualitative analysis of population data	8,678,134 insured people		Increase in treatment costs per person (in euros) among the 15–34 age group. Increase in the use of ambulatory physician services (treatment cases) among 30–34‐year‐olds. The report offers comprehensive evidence from Germany on primary care utilisation, with notable signals of rising use among younger age groups.	1
				Insured persons of the BARMER insurance company					
14	Hadwiger, M.; König, H.‐H.; Hajek, A.	Determinants of Frequent Attendance of Outpatient Physicians: A Longitudinal Analysis Using the German Socio‐Economic Panel (GSOEP)	2019	Germany	Quantiative survey	28,574 observations in outpatient physician practices (> 17 years old)		Partially informative for RQ2: The study shows that declines in physical and mental health and changes in employment/partnership status are associated with higher outpatient attendance, while a small copayment (practice fee) reduced odds—suggesting need factors dominate utilisation patterns, with some sensitivity to access barriers.	2
				German Socio‐Economic Panel (GSOEP)					
15	Hajek, A.; König, H.‐H.	Are perceived bad working conditions and perceived workplace bullying associated with doctor visits? Results of the nationally representative German General Social Survey	2019	Germany	Quantitative survey	1859 (18–74 years old)		Workplace bullying was associated with increased doctor visits in men, but not in women. Contrarily, time/performance pressure at work was only associated with increased doctor visits in women, but not in men. The probability of visiting the doctor for reasons of acute illness or feeling unwell increased with workplace bullying in men. The probability of visiting the doctor because of feeling unwell increased with time/performance pressure in women.	2
				Cross‐sectional German General Social Survey (ALLBUS).					
16	Hoffmann, K.; George, A.; Jirovsky, E.; et al.	Re‐examining access points to the different levels of health care: a cross‐sectional series in Austria	2019	Austria	Quantitative survey data	31,518		The study provides robust, population‐level evidence of increasing ambulatory secondary‐care use (2006/07 → 2014)—including age‐band data covering young adults—and attributes the trend to system design (no gatekeeping) rather than to measured HL, which it does not assess. For 15–34‐year‐olds, specialist and outpatient‐department use increased, and outpatient‐department use without GP also increased.	1,2
				Database of the Austrian Health Interview Surveys (ATHIS)					
17	Kraxner, R.; Dorner, T.E.; Roth, D.; et al.	Access points to different levels of health care over 13 years. Utilisation behaviour in a changing health care system. Results of a three‐wave cross‐sectional series in Austria	2024	Austria	Quantitative survey data	46,712		The study documents rising ambulatory utilisation over 13 years and provides age‐band results including 15–34‐year‐olds, plus system‐level interpretations (unregulated access; primary‐care coordination).	1,2
				Database of the Austrian Health Interview Surveys (ATHIS)					
18	Mantwill, S.; Schulz, P. J.	Low health literacy and healthcare utilisation among immigrants and non‐immigrants in Switzerland	2017	Switzerland	Quantitative survey	1146		Functional HL is directly related to healthcare utilisation. The effects might be amplified by (micro‐)cultural differences.	3
				Immigrant groups and native population in the German‐ and Italian‐speaking part of Switzerland					
19	Meynard, A.; Broers, B.; Lefebvre, D. et al.	Reasons for encounter in young people consulting a family doctor in the French speaking part of Switzerland: a cross sectional study	2015	Switzerland	Quantitative cross‐sectional study	594		First study describing reasons for encounter as stated by young people themselves in primary care in Switzerland. 95% of questionnaires contained valid data about reasons for encounter (*n* = 567). General and unspecific reasons were the most common in boys (44%) and girls (42%), followed by respiratory, musculoskeletal, dermatological and psychological reasons. Psychological reasons were more frequent in girls attending urban practices; musculoskeletal and dermatological reasons were more frequent in rural areas. Sexually transmitted infections or substance use were very rarely stated as a reason for encounter.	2
				Consecutive patients aged between 15 and 24 years in primary care practices in the French‐speaking part of Switzerland					
20	Neumann, A.; König, H.‐H.; Hajek, A.	Determinants of Patient Use of Telemental Health Services: Representative Cross‐Sectional Survey From Germany	2025	Germany	Quantitative cross‐sectional online survey	2082		Younger age, more favourable patient attitudes toward telemental health, more positive provider attitudes, and greater provider skills were all linked to higher patient use of telemental health services. These associations were observed specifically in the context of service uptake since the onset of the COVID‐19 pandemic.	2
				Individuals (18–74 years) who had been using mental health services					
21	OBSAN, Peter, C.; Diebold, M.; Delgrande Jordan, M.; Dratva, J. et la.	*Original title in German:* Gesundheit in der Schweiz‐ Kinder, Jugendliche und junge Erwachsene *Translated title in English:* Health in Switzerland – children, adolescents, and young adults	2020	Switzerland	Report	—		This report provides Swiss data on health service utilisation across age groups, including young adults and shows an increasing trend in ambulatory doctor visits. Aspects such as menta health, transitional care and system‐level factors are discussed as possible drivers of utilisation. Correlations between HL and utilisation are reported, though not always specific to young adults.	1,2,3
				GP consultations					
22	Rachamin, Y.; Jäger, L.; Schweighoffer, R.; Signorell, A.; et al.	The Impact of COVID‐19 on Mental Healthcare Utilisation in Switzerland Was Strongest Among Young Females‐Retrospective Study in 2018–2020	2023	Switzerland	Quantitative retrospective cohort study	—		The study observed distinct patterns for young people, most strikingly, an increase in mental healthcare utilisation among females aged < 20 years.	1,2
				Nationwide hospital data and claims data from a large Swiss health insurer					
23	Rüegg, R., & Abel, T.	The relationship between health literacy and health outcomes among male young adults: exploring confounding effects using decomposition analysis	2019	Switzerland	Quantitative survey	5959 (18–25 years old)		The study reports correlations between HL and health outcomes/behaviours in young adults, however, not directly on utilisation.	3
				Young Adult Survey Switzerland, sample was collected during recruitment for compulsory military service					
24	Stocker, D.; Jäggi, J.; Berset, M.; et al.	*Original title in German:* Junge Erwerbstätige – Arbeitsbedingungen und Gesundheit *Translated title in English:* Young workers – working conditions and health	2021	Switzerland	Trendreport; mixed‐method	—		High‐quality context on Swiss young workers' health and work environment – notably greater mental health strain, higher physical loads, poorer autonomy, strong peer/supervisor support, highest Job‐Stress‐Index imbalance, and more accidents.	4
				Young workers in Switzerland					
25	Tzogiou, C.; Boes, S.; Brunner, B.	What explains the inequalities in health care utilisation between immigrants and non‐migrants in Switzerland?	2021	Switzerland	Quantitative health survey	19,991 (17–73 years old)		Immigrants were less likely to visit a doctor than Swiss non‐migrants (e.g., doctor visit probability 74% vs. 78%). Lower doctor use by immigrants may reflect barriers in access (language, lack of knowledge of the Swiss system, out‐of‐pocket costs). Higher use of emergency departments may result from inadequate access to primary care and financial constraints (lower incomes + high out‐of‐pocket burden in Switzerland). Authors highlight HL deficits indirectly: immigrants more often did not understand doctors' information or their own insurance plan/deductible.	3
				Immigrants and non‐ immigrants in Switzerland					

### 3.2 Analytical Synthesis—Identified Themes Along the Four RQs

The following section presents an analytical synthesis of the data, organised according to the four RQs. For each RQ, relevant themes (italicised) were identified during the coding process in MAXQDA.

**RQ1:** What studies are available to date that have investigated healthcare utilisation by young adults in Switzerland, Germany and Austria?

The findings show that research on HL as a potential determinant of healthcare use among young adults is scarce and that only few studies exist to date that have investigated healthcare utilisation by young adults in Switzerland, Germany and Austria. In particular, there are few studies on primary care (even though, as noted in the introduction, the pressure is greatest in this area), and the available data is also scarce.

### 3.3 Overview of Existing Studies

Overall, the available literature on healthcare utilisation among young adults in Switzerland, Germany, and Austria was limited and heterogeneous. Rather than providing a comprehensive picture of healthcare utilisation, the identified studies focused on specific aspects of service use. Among the identified records, three were peer‐reviewed studies [28, 29, 30], three were national or insurance‐based reports/survey analyses [1, 31, 32], and one was a professional association report [3]. Six of the seven records contained age‐specific evidence relevant to young adults, while the FMH report provided only contextual, all‐age information. Four records concerned Switzerland: the Swiss Health Survey quantified general‐practitioner consultation frequency among young adults aged 15–24 and 25–34 years (Quelle); the Obsan report described physician consultations by specialty, psychiatric care, hospital outpatient and emergency‐department use, and hospitalisations among people up to age 25 (Quelle); and the research group of Rachamin et al. (2023) investigated psychiatric inpatient admissions, outpatient psychotherapy, teleconsultations, and psychotropic medication use among people aged < 20 and 20–30 years during the COVID‐19 pandemic [28].

Regarding evidence from Austria, Hoffmann et al. examined general‐practitioner, specialist, hospital outpatient, emergency and inpatient utilisation among 15–34‐year‐olds in 2006/07 and 2014, including secondary‐care use without a GP consultation [29]. Kraxner et al. examined the same utilisation outcomes and extended the observation period to 2019. These two publications partly used the same Austrian Health Interview Survey data and should therefore not be regarded as completely independent evidence. For Germany, the BARMER report provided age‐stratified routine data on ambulatory physician care and the use of digital health applications, including the age groups 18–24 and 25–34 years, although young adults were not the report's exclusive focus [32].

### 3.4 General Trends in Utilisation

The analysed literature demonstrates a clear increase in healthcare utilisation among young adults (18–35 years) in primary care and other ambulatory settings—especially across Germany and Austria, with limited but suggestive evidence for Switzerland [1, 3, 30, 31, 32].

Across Switzerland, Germany and Austria, studies report rising contacts of young adults with primary care and other ambulatory settings services in recent years: Hoffmann et al. note a significant increase in specialist and outpatient‐department use among 15–34‐year‐olds in Switzerland, with a particularly strong rise in GP visits [29]. These observations are confirmed by the Swiss Medical Association (FMH) [3] and the Swiss Federal Statistical Office (FSO), which report an increase in consultations of young adults as well [1]. In its survey on the use of the healthcare system/consultation of doctors, the FSO concludes that the number of consultations of GPs in the last 12 months increased for the age groups of 15–24‐year‐olds as well as for the 25–34‐year‐olds. In 2012, 39.3% of 15–24‐year‐olds and 35.1% of the 25–34‐year‐olds had one to two consultations with GPs, while 14.1% of the 15–24‐year‐olds and 12.2% of the 25–34‐year‐olds had three to four consultations. By 2022, these percentages increased to 42.9% and 41.5%, respectively. Data also shows that in particular young men aged 25–34 years have consulted GPs more frequently in recent years (see Table 1). Similarly, data from Austria shows that the prevalence of primary care contacts of 15–34‐year‐olds has markedly risen since 2006/07 [30]. Kraxner et al. reported that in 2019, 76.9% of young adults had at least one GP contact (+ 5.4% compared to 2006/07), 68.2% consulted a specialist (+ 6.2%), and 25.2% used outpatient‐departments (+ 6.0%), underlining a steady increase in utilisation [30]. In Germany, studies also observed a rising trend in GP visits among young adults, further substantiating the upward trajectory in care use [10, 11, 32].

### 3.5 COVID‐19 Pandemic Effects

Despite these long‐term increases, healthcare utilisation among young adults temporarily decreased at the onset of the COVID‐19 pandemic [33]. A cross‐sectional study in German primary care highlights that in the beginning of the COVID‐19 pandemic, patients avoided primary care, particularly primary care practices, (as recommended) due to infection concerns [33]. During phases of high infection risk, young adults seemed more likely to defer care, seeking treatment only for more severe conditions [33]. The Barmer Report (2022) attributes sharp increases in consultations in specialties such as Emergency Departements (EDs) and laboratory medicine to a resurgence of respiratory infections following the easing of COVID‐19 pandemic protective measures [32]. Although not youth‐specific, these dynamics disproportionately affected younger populations due to higher exposure in schools, workplaces, and social contexts. Bohlken et al. similarly points to the interplay between pandemic waves and diagnostic trends, with younger adults showing rising rates of anxiety diagnoses as the pandemic wore on [33] (Table 3).

**RQ2:** What causes for the increase in healthcare utilisation by young adults in Switzerland, Germany and Austria are discussed in the literature?

**Table 3:** Number of consultations of generalists* in the last 12 months [1].

2012, in % of the population aged 15 and older in private households														
		No consultation		1 to 2 consultation(s)		3 to 4 consultations		5 to 9 consultations		10 consultations and more		Sample		Population
		in %	VI +/− in %	in %	VI +/− in %	in %	VI +/− in %	in %	VI +/− in %	in %	VI +/− in %	*n*	in %	*N*
	**Total**	**33.5**	**0.8**	**37.6**	**0.8**	**15.6**	**0.6**	**8.2**	**0.5**	**5.2**	**0.4**	**20,807**	**100**	**6,838,268**
**Age**														
2012	15‐24–years old	34.1	2.2	39.3	2.2	14.1	1.6	8.9	1.3	3.5	0.8	2891	100	895,674
	25–34‐years old	45.2	2.5	35.1	2.4	12.2	1.8	4.9	1.0	2.7	0.8	2523	100	1,070,448
2022	15–24‐years old	27.6	2.5	**42.9**	2.7	**18.0**	2.1	8.6	1.5	3.0	0.9	2069	100	804,408
	25–34‐years old	36.4	2.5	**41.5**	2.5	**13.0**	1.7	5.2	1.1	3.8	0.9	2117	100	1,110,406
**Sex and age group**														
**Men**														
2012	15–24‐years old	36.8	3.2	40.3	3.2	12.6	2.0	8.0	1.9	2.4	0.9	1469	100	455,962
	25–34‐years old	49.8	3.8	35.2	3.6	9.9	2.7	3.2	1.3	(1.8)	(0.9)	1203	100	542,052
2022	15–24‐years old	32.9	3.8	**41.2**	3.8	**16.4**	2.8	7.3	2.2	(2.2)	(1.1)	1010	100	416,370
	25–34‐years old	39.4	3.6	**40.8**	3.6	**11.5**	2.4	**4.4**	1.5	3.9	1.4	1015	100	582,788
**Women**														
2012	15–24‐years old	31.4	3.1	38.4	3.1	15.7	2.4	9.8	1.8	4.6	1.4	1422	100	439,713
	25–34‐years old	40.3	3.4	35.0	3.2	14.6	2.5	6.5	1.6	3.5	1.4	1320	100	528,396
2022	15–24‐years old	21.9	3.0	44.8	3.7	19.7	3.0	9.9	2.2	3.8	1.5	1059	100	388,038
	25–34‐years old	33.1	3.3	42.3	3.5	14.8	2.5	6.1	1.6	3.7	1.2	1102	100	527,619

*Note:* VI +/−: Limits of the 95% confidence interval. They indicate the range in which the actual value is located with a 95% probability. (): Insufficient number of observations (< 30). They indicate limited statistical representativeness or a lack of statistical reliability.

*Source:* FSO ‐ Swiss Health Survey, © FSO.

* With GP and/or family doctor.

A broad set of individual, social, and systemic factors are analysed and suggested as underlying causes for the general rise in healthcare‐seeking behaviour among young adults in Switzerland, Germany and Austria. These range from mental health burdens and HL limitations to socioeconomic inequalities and structural characteristics of healthcare systems. Pandemic dynamics have further reinforced and reshaped utilisation patterns.

### 3.6 Mental Health and Psycho‐Social Stressors

Young adults face particular psychosocial challenges that appear to driving use of healthcare services [31, 34]. Amstad et al. point to the increasing prevalence of mental health issues, with about one in five young adults experiencing a psychological crisis or disorder by the age of 25 [34]. During the COVID‐19 pandemic and including its aftermath, mental health burdens were especially pronounced among younger and female individuals with limited social and economic resources and greater exposure to stressors such as job loss or loneliness [33]. Bohlken et al. shows that while diagnoses dropped sharply during the first lockdown—largely due to infection fears and avoidance of GP visits—subsequent quarters of 2020 saw significant increases in diagnoses of anxiety disorders. Complementary evidence from Switzerland highlights how younger workers reported relatively good physical health compared to older groups but at the same time experience higher levels of stress, emotional exhaustion, depressive symptoms, and suicidal thoughts [35]. They also exhibited lower self‐efficacy and coping skills, which contributed to elevated demands for medical support. Work‐related exposures, limited autonomy, and spillover between private life and work further amplified this imbalance, as captured by the so called ‘Job‐Stress‐Index’, which was most unfavourable among the youngest age groups [35].

### 3.7 Sociodemographic Factors

Social status, education level, and migration background emerged as cross‐cutting drivers of healthcare utilisation [36]. Young adults with fewer social and economic resources were more likely to experience stress and mental health burdens, which in turn raised healthcare needs [7, 37]. Structural inequalities, such as lower coping resources and higher exposure to precarious employment, further reinforced these trends [35]. Getting married and changes to non‐working were both associated with an increased likelihood of frequent attendance in an identified German study on determinants of frequent attendance of GPs [38]. According to this study, one possible explanation for the increased likelihood of becoming a frequent user of healthcare services after marriage is greater awareness of one's personal health, fostered by the additional health‐related advice and support provided by a spouse [38]. In addition, Mantwill and Schulz present in their Swiss study that immigration and language background strongly predict overutilization [36].

### 3.8 System‐Level and Structural Factors

Giezendanner et al. discusses how primary care capacity, specialist density, and the absence of strict gatekeeping in Germany and Austria shape utilisation levels [39]. Furthermore, free provider choice and supply‐sensitive consumption are seen as leading to higher contact rates with GP practices. Kraxner et al. also note that in Austria, easy access to secondary outpatient services encourages direct specialist use [30]. Short GP consultation times and limited coordination functions may even increase onward referrals, with difference‐in‐differences estimates showing that young adults who first saw a GP were ~19% more likely to also see a specialist [30]. Frese et al. (2011) highlight how parallel access to paediatricians in Germany contributes to different consultation patterns among adolescents, especially for conditions such as respiratory, digestive, and dermatological complaints [40]. According to the study, this structural feature might partly explain why young people in Germany consult GP practices more often than young adults of other European healthcare systems [40].

**RQ3:** What correlations between HL and the utilisation of the healthcare system by the general population in Switzerland, Germany and Austria are reported in the literature?

HL is consistently identified as a critical explanatory factor, though the strength and direction of the associations vary depending on the HL dimension measured, the type of healthcare service, and contextual factors.

### 3.9 Low HL and Higher Frequency of Healthcare Utilisation

Multiple studies document that individuals with limited HL tend to consult physicians more frequently: De Gani et al. demonstrates that low HL is associated with greater difficulties in dealing with health‐related information, potentially leading to inefficient or higher healthcare utilisation [7]. Also Franzen et al. shows that lower HL significantly correlated with more GP visits, even after controlling for sociodemographic and health‐related factors, and these associations held across 2 years of data [37].

### 3.10 Low HL and Higher Healthcare Costs

The correlation between HL and healthcare utilisation is also reflected in healthcare costs. Franzen et al. report that lower HL was linked to higher total healthcare costs and higher outpatient costs [37]. While trends for inpatient costs and hospital days pointed in the same direction, they did not reach statistical significance [37]. The more recent study of Gerich et al. further shows that high HL combined with high health knowledge is associated with fewer doctor visits and lower medication costs, whereas high HL combined with low knowledge is linked to more doctor visits and rising costs with age [41]. Their study highlights that HL interacts with knowledge in shaping utilisation patterns and costs.

### 3.11 Low HL and Over‐/Underutilisation of Healthcare

Some studies emphasise the dual risk of inappropriate healthcare use. Mantwill and Schulz emphasise that higher functional HL (measured by S‐TOFHLA) is significantly associated with less underutilisation of necessary care for serious health issues. However, paradoxically, after adjustment, higher functional HL is also linked to more overutilization for minor symptoms, potentially reflecting greater health consciousness and a tendency to seek reassurance [36].

### 3.12 HL and Health Inequities in Healthcare Utilisation

HL correlates not only with service use levels but also with inequities in access and experiences. Tzogiou reports that immigrants with lower HL proxies—such as difficulties communicating with doctors or understanding insurance details—are more likely to experience utilisation gaps, especially regarding insurance coverage and service access [42]. Berens et al. also underlines that low HL correlated with sociodemographic factors such as low social status and migration background, both of which are associated with higher utilisation patterns [43].

**RQ4:** Are there studies that have already investigated the role of HL on healthcare utilisation by young adults in Switzerland, Germany and Austria?

The direct investigation of HL as a driver of the increase in healthcare utilisation among young adults in Switzerland remains limited. Nevertheless, two key studies (one from Germany and one from Austria) explicitly address this question and provide initial evidence of HL as a meaningful explanatory factor.

In Germany, Berens et al. find that young adults with limited HL are substantially more likely to report multiple doctor visits, with more than 70% of high‐frequency users being at special risk of limited HL [43]. Having more than two doctor visits in the past 12 months was significantly associated with lower HL across all age groups. Two years later, the same research group confirmed these findings: respondents with lower HL scores reported more frequent use of all curative health services [44]. Regression analyses shows HL remaining a significant independent predictor of GP visits (*β* = –0.066) and visits to other health professionals (*β* = –0.103), though no significant effect was found for hospital or emergency service use [44]. This association may reflect the increasing number and complexity of health problems during this phase of life, often described as the ‘rush hour of life’, in which young adults face simultaneous demands from work, family, and community. The findings further suggest that limited HL might amplify the need for professional support when young adults are navigating multiple stressors and health‐related decisions, thereby contributing to higher utilisation.

In Austria, Gerich et al. offer more direct evidence by explicitly modelling HL as a moderator of the age‐utilisation relationship [41]. The study shows that the level of HL significantly shapes trajectories of healthcare use: the effect of age on doctor visits and medication costs was strongest among individuals with lower HL and weakest among those with higher HL. This moderation effect indicates that HL not only correlates with utilisation but also helps explain interindividual differences in how healthcare use increases with age. Thus, HL seems to function as a key mechanism influencing utilisation dynamics over time [41].

## 4 Discussion

Overall, the identified evidence was mainly cross‐sectional and focused on selected forms of healthcare utilisation rather than on the relationship between health literacy and utilisation over time. Longitudinal and intervention studies were largely absent, and direct evidence for young adults aged 18–35 years remains limited. Nevertheless, across the four RQs, a coherent picture emerges:

### 4.1 Healthcare Utilisation Is Increasing Among Young Adults

The available evidence shows a clear trend in increasing healthcare utilisation among young adults in Switzerland, Germany and Austria, particularly for GP visits, specialist consultations, and primary care use [1, 3, 32]. These increases are well‐documented in both longitudinal survey [1] and insurance data [32]. Temporary declines during COVID‐19 pandemic highlight the sensitivity of utilisation to external events [33], but the overall trajectory points upward.

Beyond utilisation rates, evidence also sheds light on how young adults perceive primary care. Tille (2019) found that young adults (aged 18–34) in Germany more often rated GP responsiveness poorly compared to older adults [45]. Responsiveness in this context indicates the ability of a health service to meet the population's legitimate expectations regarding non‐medical and non‐financial aspects of the care process [45, 46]. Deficits were particularly pronounced in domains such as trust (OR 2.59, 95% CI 1.34–5.01) and confidentiality (OR 2.34, 95% CI 1.74–3.16). This indicates not only increased utilisation but also a distinct pattern of patient experiences that may influence ongoing care‐seeking behaviours [45].

### 4.2 The Causes for an Increased Healthcare Utilisation Are Layered

The causes for these increases are multifaceted and multi‐layered [3, 16, 47, 48, 49]. Individual‐level drivers include higher prevalence of mental health issues in young adulthood, psychosocial stressors (intensified during the COVID‐19 pandemic), and less coping resources. Limited HL and socioeconomic inequalities also play a significant role, as do migration background and education [50]. Structural and systemic factors, such as free provider choice, specialist density, short GP consultation times, and weak gatekeeping further shape utilisation patterns. Pandemic‐related epidemiological shifts, especially waves of respiratory illness, additionally contribute to spikes in demand [33]. These patterns also illustrate how prolonged social isolation, restrictions, home‐office pressures, and childcare responsibilities can intensify mental strain, particularly for younger adults [33, 51]. These determinants reflect structural inequities that can contribute to greater utilisation among specific youth populations, even if they do not directly explain increases in doctors' visits over time [36]. A further, however, not age‐specific, notable aspect mentioned in the identified literature is work related stress. While workplace bullying was associated with increased GP visits in men, time or performance pressure at work was primarily associated with increased GP visits in women [52]. Moreover, in a Norwegian study, Le et al. identify financial deprivation (next to low HL) as important correlates of more frequent GP visits. They emphasise that financially deprived young people have significantly lower HL scores, which were also linked to more GP visits [53]. In addition, in the German longitudinal analysis study conducted by Hadwinger et al., both marriage and transitions into non‐employment were associated with a higher likelihood of frequent physician visits [38]. This association between employment status and frequent attendance extends previous research, which has often relied on cross‐sectional designs [54, 55]. One possible explanation for the increased tendency to become a frequent attender after marriage could be greater awareness of one's personal health, potentially fostered by the health‐related advice and support provided by one's spouse [38].

### 4.3 HL as a Central Resource

The results of this scoping review emphasise that HL is a central determinant, both as a correlate of healthcare utilisation and as a moderator shaping how strongly utilisation rises over time [37]. A consistent body of evidence shows that limited HL is associated with more frequent healthcare use, especially GP visits, and higher costs [7, 56, 57]. These findings are in line with other European insights on the correlation of HL and healthcare utilisation [53, 58]. Functional HL protects against underutilisation of necessary care, but paradoxically may also be linked to greater overutilization of minor services, likely reflecting proactive health‐seeking among health‐conscious young adults [36]. These associations hold across Switzerland, Germany, and Austria, with regional variations (e.g., Swiss Italian‐speaking regions). Migration background and social status further moderate HL utilisation relationships.

Direct evidence on low HL as a driver of increasing healthcare utilisation is limited but emerging in recent years [53, 59]. German findings [43] suggest that young adults with frequent doctor visits more often report limited HL. Austrian evidence [41] demonstrates that HL moderates the age‐healthcare‐utilisation relationship: individuals with lower HL show steeper increases in doctor visits and medication costs as they are getting older, compared to peers with higher HL. Together, these studies indicate that HL not only correlates with utilisation levels (see RQ3) but (limited) HL of young adults might also help to explain the trend of increased healthcare utilisation among young adults (see RQ1–RQ2).

In addition to individual HL, OHL may influence healthcare utilisation by shaping how easily individuals can access, navigate, and use healthcare services. Organisational barriers, such as complex appointment systems, fragmented information, or poorly designed digital communication, may contribute to repeated or avoidable healthcare contacts, particularly among young adults with limited health literacy. Strengthening OHL could therefore complement individual HL interventions and support more needs‐based healthcare utilisation [23].

Thus, despite promising new insights on the matter, research gaps remain, particularly for Switzerland, where evidence is still limited. Research also lacks in establishing causal pathways linking HL to healthcare utilisation patterns in young adults aged 18–35, but also generally. Overall, the findings underscore the importance of researching, addressing and improving HL proactively and systematically in young adulthood. In addition, future research should emphasise not only how to address and improve HL in young adults, but also how OHL indicators can be strengthened to develop an OHL friendly ecosystem and a youth friendly, HL responsive healthcare setting. Doing so may not only support better decision‐making and self‐efficacy of young adults, but also help mitigating uncertainty, as well as unnecessary increases in healthcare use while ensuring that serious conditions are appropriately treated.

## 5 Limitations

As with most scoping reviews, this study is limited by its broad scope and reliance on available published evidence. The heterogeneity of study designs and contexts of the identified studies complicates direct comparisons. Thus, the findings should be interpreted as descriptive rather than causal. In addition, despite comprehensive searching in various databases, some relevant studies may have been missed, and the evidence base only reflects the situation up to July 2025.

## 6 Conclusion

This scoping review highlights that healthcare utilisation among young adults is increasing in Germany and Austria, with suggestive but less developed evidence for Switzerland. Multiple interrelated factors contribute to this trend. On the individual level, mental health burdens, life‐phase stressors, and limited HL are central drivers. At the social level, socioeconomic inequalities, migration background, and varying coping resources amplify these demands. System‐level characteristics, such as specialist density, free provider choice, and weak gatekeeping further shape pathways and use of care. Superimposed on these dynamics, pandemic‐related disruptions and epidemiological shifts (e.g., respiratory infections) can act as accelerants of utilisation. Across the three countries, HL emerges as a critical explanatory factor. The evidence consistently shows that limited HL is associated with more frequent healthcare use, higher costs, and a greater likelihood of both over‐ and underutilisation. Importantly, HL does not only correlate with current utilisation but also moderates how strongly healthcare use increases with age, particularly during young adulthood. Studies from Germany and Austria demonstrate that low HL magnifies upward utilisation trajectories, while higher HL appears protective.

Despite these insights, notable gaps remain. First, research in Switzerland is comparatively limited, especially regarding temporal trends and causal analyses linking HL to utilisation in young adulthood. Second, most available studies are cross‐sectional, limiting causal inferences. Third, the interplay between HL and system‐level structures (e.g., insurance design, provider availability, regional differences) remains underexplored. Addressing these gaps in the future will be essential to develop a more comprehensive understanding of why young adults increasingly use healthcare services and how they can be effectively supported in health‐related decisions. Future research should also examine how individual HL interacts with organisational factors, such as the accessibility of services, navigation processes, and the clarity of digital and non‐digital communication, and whether improvements in individual or organisational health literacy support more needs‐based healthcare utilisation. This in turn will support healthcare providers, leading to more efficient and effective healthcare use and dampen the steady increase of the healthcare system's burden and costs.

## Author Contributions

**Anna‐Sophia Beese:** conceptualisation, methodology, project administration, writing – review and editing, writing – original draft, data curation, formal analysis. **Valentin Leu:** conceptualisation, writing – original draft, writing – review and editing, methodology, data curation, formal analysis. **Oliver Senn:** conceptualisation, supervision, writing – review and editing. **Saskia Maria De Gani:** writing – review and editing, conceptualisation, supervision.

## Funding

The authors have nothing to report.

## Ethics Statement

The study was conducted according to the guidelines of the Declaration of Helsinki. Ethical review and approval were waived for this study, as it is outside the scope of the Human Research Act and did not require approval by the Swiss Association of Research Ethics Committees (BASEC‐Nr. Req‐2021‐01325). All included studies were publicly available, and no ethical approval was required. The research process adhered to the principles of good scientific practice, and the sources were selected, analysed, and reported with academic integrity.

## Conflicts of Interest

The authors declare no conflicts of interest.

## Supporting information


Supporting File

## Data Availability

The detailed search strategy is available as a supplementary file.
