# Supplementary material for: Health Literacy as a Potential Determinant of Healthcare Use Among Young Adults in German‐Speaking Countries With a Special Emphasis on Primary Care: A Scoping Review
Source: Health Expect. 2026 Nov 4;29(6):e70945. doi: 10.1111/hex.70945 (PMC13732647; doi:10.1111/hex.70945)
Supplement: Supplementary file 1 [file HEX-29-e70945-s001.docx]

|  | **RQ1:** What studies are available to date that have investigated healthcare utilization in primary care by young adults in Switzerland, Germany and Austria? | **RQ2**: What causes for the increase in healthcare utilization by young adults in Switzerland, Germany and Austria are discussed in the literature? | **RQ3:** What correlations between HL and the utilization of the healthcare system by the general population in Switzerland, Germany and Austria are reported in the literature? | **RQ4:** Are there studies that have already investigated the role of HL on healthcare utilization by young adults in Switzerland, Germany and Austria? |
| --- | --- | --- | --- | --- |
| Search terms | (("Health* utiliz*" OR "health service use" OR "healthcare use" OR "doctor visit*" OR "visit* to GP" OR "number of visit*" OR frequen*)  OR ("Health Services/utilization"[MeSH] OR "Health Services Needs and Demand"[MeSH]))  AND  ("Switzerland" OR German* OR Swiss OR Austria*)  AND  ("young adults" OR adolescents OR youth OR young OR "young people" OR "Young Adult"[MeSH] OR "Adolescent"[MeSH])  AND  (ambulatory OR "primary care" OR "outpatient care" OR "general practice" OR "family practice" OR "outpatient services" OR "ambulatory care facilities")  AND  (increase OR rise OR growth OR trend*) | ("Healthcare utilization" OR "doctor visits" OR "number of visits")  AND  ("Switzerland" OR German* OR Swiss OR Austria*)  AND  ("young adults" OR adolescents OR youth OR young)  AND  (causes OR reasons OR factors OR determinants)  AND  (increase OR rise OR growth OR trend*) | (("healthcare utilization" OR "healthcare use" OR "health service use" OR "doctor visits" OR "number of visits" OR "Health Services/utilization"[MeSH] OR "Health Services Needs and Demand"[MeSH]))  AND  ("Switzerland" OR German* OR Swiss OR Austria*)  AND  ("young adults" OR adolescents OR youth OR young OR "young people" OR "Young Adult"[MeSH] OR "Adolescent"[MeSH])  AND  ("health literacy" OR "medical literacy" OR competenc* OR correlat* OR determinan* OR "Health Literacy"[MeSH]) | (("healthcare utilization" OR "healthcare use" OR "health service use" OR "doctor visits" OR "number of visits" OR "Health Services/utilization"[MeSH] OR "Health Services Needs and Demand"[MeSH]))  AND  ("Switzerland" OR German* OR Swiss OR Austria*)  AND  ("young adults" OR adolescents OR youth OR young OR "young people" OR "Young Adult"[MeSH] OR "Adolescent"[MeSH])  AND  (increas* OR rise OR grow* OR trend*)  AND  ("medical literacy" OR competenc* OR "Health Literacy"[MeSH]) |
| Application to | Title, abstract, keywords, free full text | Title, abstract, keywords, free full text | Title, abstract, keywords, free full text | Title, abstract, keywords, free full text |
